# Expression of *Brugmansia candida *Hyoscyamine 6beta-Hydroxylase gene in *Saccharomyces cerevisiae *and its potential use as biocatalyst

**DOI:** 10.1186/1475-2859-7-17

**Published:** 2008-05-27

**Authors:** Alejandra B Cardillo, Julián Rodríguez Talou, Ana M Giulietti

**Affiliations:** 1 Microbiología Industrial y Biotecnología, Facultad de Farmacia y Bioquímica, Universidad de Buenos Aires, Junín 956 6° piso (1113), Buenos Aires, Argentina

## Abstract

**Background:**

Tropane alkaloids, mainly hyoscyamine and scopolamine, are widely used in medicine due to their anticholinergic activity. Scopolamine has a higher demand being the more valuable alkaloid due to its fewer side effects and higher physiological activity. Anisodamine (6β-hydroxyhyoscyamine) is the intermediate in the conversion of hyoscyamine into scopolamine. Current studies report that this alkaloid is potentially applicable in medicine. The gene that codifies for Hyoscyamine 6-β hydroxylase, the enzyme responsible for hyoscyamine hydroxylation and epoxidation, leading to scopolamine was isolated from *Brugmansia candida*.

**Results:**

The *h6h*cDNA was cloned into pYES2.1 and pYES2.1/V5-His-TOPO vectors to produce an untagged and a tagged protein, respectively. The H6H enzyme was produced in *Saccharomyces cerevisiae *in order to obtain a biological catalyst for potential industrial applications. Protein extracts of the induced yeast were analyzed by Western blot. The expression was detected 4 h after induction and no degradation was observed during the period assayed. The tagged and the untagged proteins were able to transform hyoscyamine, showing a functional expression of the *h6h*cDNA.

**Conclusion:**

The strains obtained in this work are promising and potentially applicable in biocatalytic processes.

## Background

The tropane alkaloids hyoscyamine and scopolamine are widely used as pharmaceuticals due to their anticholinergic activity. The application of scopolamine in medicine is preferred because of the fewer side effects produced and higher physiological activity of this alkaloid. Anisodamine, other tropane derivative, also presents pharmacological properties, but is less potent and less toxic than the other compounds. Anisodamine offers additional therapeutic applications such as the treatment of septic shock, circulatory disorders, gastric ulcers, respiratory diseases, gastrointestinal colic, organophosphorus poisoning, migraine, acute glomerular nephritis, eclampsia and rheumatoid arthritis [1].

**Figure 1:**
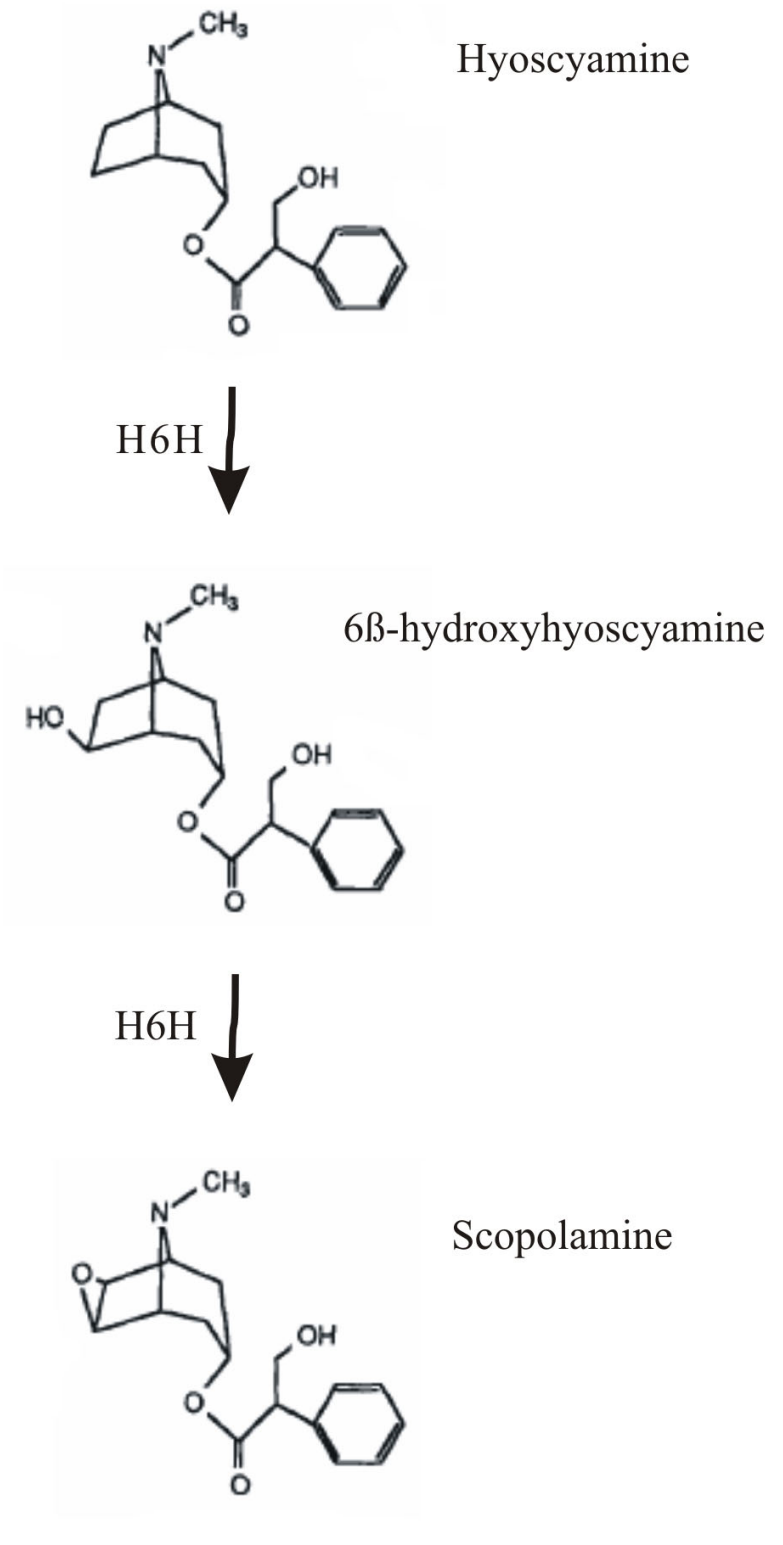
Reactions catalyzed by H6H leading to 6β-hydroxyhyoscyamine and scopolamine.

Fig. 1 shows the chemical structures of hyoscyamine, anisodamine and scopolamine. Hyoscyamine 6-β hydroxylase (H6H, EC 1.14.11.11), a plant enzyme, catalyses the hyosacyamine hydroxylation producing 6β-hydroxyhyoscyamine (anisodamine) and then its epoxidation leading to scopolamine (6,7-β-epoxide of hyoscyamine).

Scopolamine is the most valuable, with a 10 times higher commercial demand than that of hyoscyamine [2-7]. For this reason the transformation processes is an attractive strategy for anisodamine and scopolamine production. Among the possible transformation processes, the bioconversion appears to be the preferable choice. The first step in this direction is having an efficient system for recombinant enzyme production. From this point of view, *Saccharomyces cerevisiae *is a good candidate. This yeast has been used for many biotechnological applications including the expression of recombinant proteins [8]. It has also the ability to perform eukaryotic modifications and appropriate folding with the advantage of the easy manipulation and the rapid growth usual in prokaryotic cells [9]. In addition, *S. cerevisiae *is not pathogenic and has been classified as GRAS (Generally Regarded as Safe)[10]. The choice of this microorganism is also based on the availability of a well known culture technology with an easy scale-up.

The aim of this work is to obtain a S. *cerevisiae *strain harbouring the H6H plant enzyme for catalytic applications in the production of scopolamine and/or 6β-hydroxyhyoscyamine. For this propose it is necessary to isolate the *h6h *gene as a first step. *Brugmansia candida *(*syn Datura candida*) a tropane alkaloid producer plant [11], is a suitable source for the isolation of *h6h *gene. The second step is cloning this gene in *S. cerevisiae *and assesing the capacity of the transgenic yeast to produce catalytically active plant H6H.

## Results

### Cloning of *h6h*cDNA

The amplification of the *h6h *cDNA was carried out by PCR from total RNA preparations obtained from *B. candida *immature anthers as it was reported previously[12].

cDNAs encoding full-length H6H were cloned into the yeast expression vectors pYES2.1/V5-His-TOPO and pYES2.1 containing the GAL1 promoter as described in Methods. The constructions obtained with the pYES2.1/V5-His-TOPO vector were sequenced using Gal1 and V5C primers from the TOPO cloning KIT and the pYES2.1 ones with the specific primers mentioned in Methods section. The sequences obtained for the *B. candida h6h *cDNA (GenBank: EU530633) were highly homologue to the *Hyoscyamus niger h6h *gene (Genbank: M62719)

### H6h expression in *S. cerevisiae*

**Figure 2:**
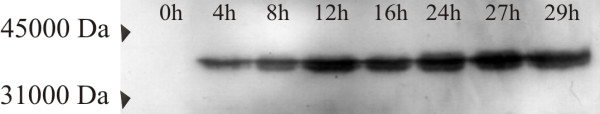
Western blot performed with cell lysates of *S. cerevisiae *producing the recombinant H6H enzyme from *B. candida *after different post-induction times: 0, 4, 8, 12, 16, 24, 27, 29 h.

According to the vector used, two *S. cerevisiae *strains were obtained. The Y_H6H 2-2 _strain carries the pH6H2-2 vector and the Y_pAC3-15(4) _strain the pAC3-15 one. The induction of the H6H expression was analyzed by sampling aliquots at different times after culture induction according to Methods. Yeast recombinant cells were lysed as described and the protein extract was analyzed by Western blot. The expression of the H6H enzyme as a fusion protein allowed the detection of the fused epitope by commercially available antibodies. The H6H enzyme was produced in the soluble form determined by Western blot. The production of the H6H enzyme was detected 4 h after induction and no degradation was observed during the time assayed (Fig. 2).

### Enzyme activity

**Figure 3:**
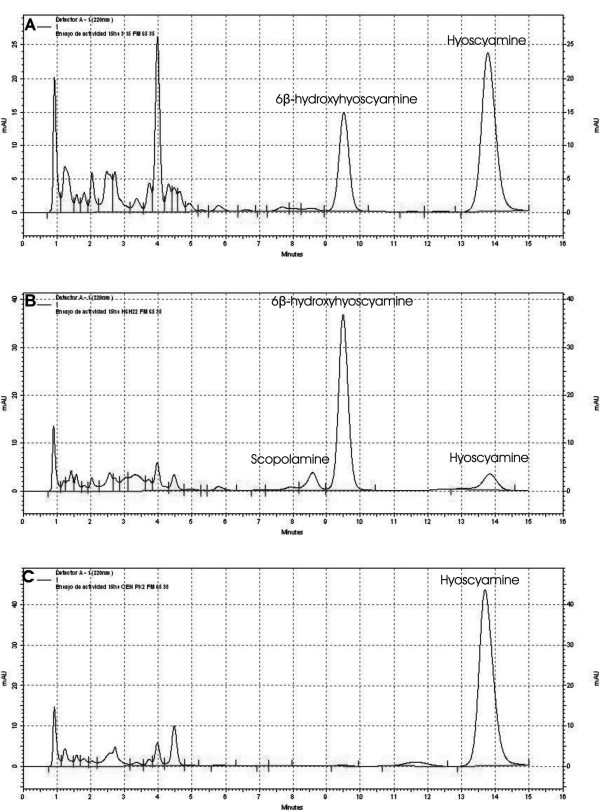
HPLC chromatograms obtained after 15 h incubation. (a) Tagged protein. (b) Untagged protein. (c) Control reaction (carried out with a crude protein preparation of wild type cells).

Different products were obtained depending on the enzyme used in the activity assay (tagged or untagged enzyme). Table 1 shows the percentages of the alkaloids produced in the activity assays. When the reaction was incubated for 2 hs, the tagged protein was able to produce a 15% of 6β-hydroxyhyoscyamine. The other 85% remained as hyoscyamine. On the other hand, the untagged protein produced a 53.7% of the intermediate in the same incubation time. Figure 3 shows the chromatograms obtained for each activity reaction at 15 h incubation. This time was arbitrarily elected and does not indicate the end of bioconversion. The reaction carried out with the tagged protein yielded on HPLC one product with a retention time of 9.5 min comparable with 6beta-hydroxyhyoscyamine (Fig. 3a). This protein was able to produce approximately a 35.7% of 6β-hydroxyhyoscyamine in 15 h of incubation. In contrast, the reaction carried out with the untagged enzyme yielded two products with retention times of 9.5 and 8.6 min on HPLC (Fig. 3b). The later corresponds to the retention time of scopolamine. In this case 83.3% of 6β-hydroxyhyoscyamine and 7.6% of scopolamine were produced. Only a 9% of hyoscyamine was not transformed by the untagged enzyme. No product formation was seen in control reactions performed with crude extract of induced wild type strain (Fig 3c). Table 2 shows the activity of the tagged and the untagged enzymes produced per mg of total proteins of the crude extract. Comparing these results to previous reports of the H6H, it can be noticed that the activity of these preparations is of the same order of magnitude and is superior to results obtained using the H6H purified from hairy root extracts [13-15].

**Table 1:** Alkaloids produced from hyoscyamine with the tagged and untagged H6H after 2 and 15 h of incubation.

	6β-hydroxyhyoscyamine (%)		Scopolamine (%)	
	
Time (h)	2	15	2	15
Untagged H6H	53.7	83.3	-	7,6
Tagged H6H	15.0	35.7	-	-
Negative control	-	-	-	-

The percentage values of 6β-hydroxyhyoscyamine and scopolamine are the means of three independent determinations which differs in no more than 10%

**Table 2:** Hydroxylase and epoxidase activities in crude protein extract of recombinant *S. cerevisiae *strains, Y_pAC3-15(4) _and Y_H6H 2-2 _overexpressing the *B. candida h6h *gene.

Crude extract	Hydroxylase activity	Epoxidase activity
	
	Specific Activity (nKat mg^-1^)	Specific Activity (nKat mg^-1^)
Untagged H6H	2.60 ± 0.19	0.24 ± 0.02
Tagged H6H	0.89 ± 0.06	ND*

*ND: Not detected

## Discussion

The *h6h *cDNA was successfully amplified from immature anthers from *B. candida *flower buttons.

Concerning to the sequences obtained, the results are coincident with previous reports about the H6H from other related species [13,14]. The 2OG-Fe (II) oxygenase superfamily domain is also conserved in the *h6h *gene from *B. candida *(GenBank: EU530633). This sequence element that is considered to be an iron binding site is also found in other 2-oxoglutarate dependent dioxygenases from plant, bacteria and fungi [15,16]. In addition, the sequence analysis shows that the *h6h *sequence has similarity to other hydroxylases including those involved in the biosynthesis of ethylene and anthocyanins[17].

Employing current methodologies of transformation of yeast it was possible to obtain a recombinant *S. cerevisiae *strain harboring the plant enzyme (H6H) with the aim to obtain the biological catalyst for the conversion of hyoscyamine.

In order to follow the production of the H6H enzyme by immunodetection with antibodies it was necessary to incorporate an epitope to the recombinant protein for the analysis.

The tagged enzyme was able to transform hyoscyamine into 6β-hydroxyhyoscyamine while the untagged protein transformed hyoscyamine into scopolamine and 6β-hydroxyhyoscyamine, showing a functional expression of the *h6h*cDNA. The activity of the enzyme produced in *S. cerevisiae *was similar to previous reports about the activity of other H6H [13,18]. The specific activity of the crude extract from yeast has values of the same order of magnitude and even higher than previous reports (see Table 1). Former publications referred the enzyme activity to purified H6H concentration. It should be kept in mind that the results reported here are referred to total protein concentration of the crude extract. The results presented in this work, showed that the epitope and the histidine tag fused to the protein reduce the ability of the enzyme to transform the alkaloid. This protein presented a lower rate of conversion of hyoscyamine than the untagged enzyme. Another advantage of the later is the ability to produce scopolamine and not just 6β-hydroxyhyoscyamine within the incubation times assayed. The differences observed between the tagged and untagged enzymes could be attributed to the flag. It is possible that the epitope and the histidine tag affect in some way the enzyme activity, modifying the correct folding of the protein.

Being scopolamine the more valuable tropane alkaloid several strategies were developed to increase its production in plant systems. Two examples of this are the overexpression of the *h6h *gene in *Hyoscyamus muticus *hairy root [19] and the overexpression of the Putrescine N-methyltransferase (PMT) and H6H in *H. niger *hairy root [4]. However, the expression of plant genes in microorganisms could be a useful and economically attractive approach for the production of plant proteins and their products. The main advantages that microorganisms have over plant systems are that the production of biomass is achieved quicker and that the genetic is well known [20]. Alkaloids are complex structures making chemical synthesis complicated and expensive. For all these reasons, microorganisms are considered an attractive alternative for the production of alkaloids. There are several reports about the microorganism production of plant enzymes involved in alkaloid biosynthetic pathways [21-24]. In addition, the production of the H6H in *Escherichia coli *for functional studies has been described [13,15].

As far as the authors are aware, this is the first report about the cloning of the *h6h *gene in *S. cerevisiae*. H6H is a key enzyme from the tropane alkaloid pathway that allows the production of scopolamine, which is a valuable alkaloid with a demand calculated 10 times higher than its precursor, hyoscyamine. In addition, this enzyme has the ability of producing anisodamine which is promissory for a therapeutic use [1].

Additionally, *S. cerevisiae *has certain advantages over *E. coli *for pharmaceutical applications. The later produces endotoxins and the use of *S. cerevisiae *is considered to be safe, having a long history of application in the alimentary and pharmaceutical industry.

## Conclusion

According to the results obtained in this work, we can conclude that the recombinant strains obtained are promising for the production of scopolamine and anisodamine by biotransformation. Further studies are being performed for the optimization of the biotransformation and future technological applications.

## Methods

### Plant material

Immature anthers (microspore mother cells) from *B. candida *flower buttons were harvested at the "Jardín Botánico" of Buenos Aires (Argentina).

### Strains and vectors

*E. coli *strain DH5α (F-*rec*A1 *end*A1 *hsd*R17 (rk-, mk+) *sup*E44 λ-*thi*-1 *gyr*A96 *rel*A1) was maintained at 37°C in Luria Bertani medium (LB) (Bacto-tryptone 10 g l^-1^, bacto-yeast extract 5 g l^-1^, NaCl 10 g l^-1^). For recombinant strains the media was supplemented with 100 μg ml^-1 ^ampicillin.

*S. cerevisiae *strain CEN PK2 (Acc. n° 30000D, Eurofan) (Mata/Matα ura3-Δ2/ura3-Δ2 trp1-289/trp1-289 leu2-3, 112/leu2-3, 112 his3Δ1/his3Δ1 mal2-8 C/mal2-8 C suc2/suc2) was kindly supplied by Dr. Susana Silverstein, IFYBINE-FCEN UBA. *S. cerevisiae *wild type strain was grown in YPD medium (10 g l^-1^yeast extract, 20 g l^-1 ^peptone, 20 g l^-1 ^glucose) at 30°C. The recombinant yeast strains were maintained in the selection medium YNBD-U (yeast nitrogen base without uracil).

The pYES2.1/V5-His-TOPO and pYES2.1 vectors from Invitrogen (California, USA) were used to insert the *h6h*cDNA according to the manufacturer's instructions.

### Chemicals

L-Hyoscyamine hydrobromide, Scopolamine hydrobromide and all the media components were purchased from Sigma Chemical Co. (St. Louis, USA). Anisodamine (6β-hydroxyhyoscyamine) was kindly provided by Dr. László Kursinszki, Semmelweis University. Budapest, Hungary. YNB medium and Catalase were purchased from from ICN Biomedicals, Inc. (California, USA), PCR reagents and the Anti V5 antibody by Invitrogen (California, USA), ECL reagent from GE (Little Chalfont Buckinghamshire, UK) and the Complete, EDTA-free Protease Inhibitor Cocktail Tablets were supplied by ROCHE (Mannheim, Germany).

### Cloning of *h6h*cDNA

The *h6h *amplified PCR product of 1000 bp was gel purified and cloned into the pYES2.1/V5-His-TOPO and pYES 2.1 vectors containing the galactose-inducible glucose-repressible GAL1 promoter. Specific primers were designed based on the sequence of the H6H gene from *H. niger *(Genbank: M62719). The resulting primers were: 5'GACATTGATGGCTACTTTTGTGTCGAACTGG3' (forward) and 5'ACGACCTTCGATATTGATTTTATATGGC3' (reverse) for the pYES2.1/V5-His-TOPO vector. The reverse primer eliminates the stop codon of the amplified sequence. For the pYES2.1 vector the resulting primers were: 5'GGGGTACCCCGTGATGGCTACTTTTGTGTCGAACTGG3' (forward) and 5'CCGCTCGAGCGGTTATTAGACATTGATTTTATATGGC3' (reverse).

In the first case the PCR product is expressed as a fusion to the C-terminal V5 epitope and poly-histidine tag. The resulting recombinant plasmid named pAC3-15 and the pH6H2-2 respectively were amplified in *E. coli *DH5α and were sequenced on both strands.

### DNA sequencing and sequence analysis

DNA sequencing was performed by Macrogen (Seoul, Korea). The results were analysed using the BLAST algorithm of the National Center for Biotechnology Information (NCBI).

### Yeast transformation and heterologous expression

*S. cerevisiae *strain CEN PK2 was used as host for the pAC3-15 and the pH6H2-2 recombinant vectors. Yeast chemical transformations were performed by the lithium acetate protocol [25]. The clones obtained were grown at 30°C on minimal medium YNBD-U supplemented with histidine, leucine and tryptophane.

Recombinant yeast was grown for 16 h into YNBD-U medium at 30°C, 200 rpm. For the H6H expression the cells were separated by centrifugation, rinsed with distilled water and cultured again in the same medium containing galactose 2% (YNBG-U media) instead of glucose. Cells were harvested at 0, 4, 8, 12, 16, 24, 27 and 29 h after induction.

### Western blot analysis

Yeast transformant cells were lysed by shear forces using acid-washed glass beads and protease inhibitor Cocktail Tablets from Roche. The extent of lysis was checked with small aliquots under the microscope. Proteins were measured by the Bradford method [26]. Aliquots of 50 μg of protein lysate were loaded into a 12% poliacrylamide gel. SDS-PAGE and Western blot were performed according to the protocol described by Laemmli [27]. The protein was revealed by chemiluminescent ECL reagents.

### Enzyme activity assay

Crude preparations of the recombinant H6H were assayed for the enzymatic activity by measuring the transformation of hyoscyamine. The 10 ml reaction mixture contained 50 mM Tris-HCl buffer (pH7.8 at 30°C), 4 mM sodium ascorbate, 0.4 mM FeSO_4_, 1 mM 2-oxoglutarate, 0.2 mM *l*-hyoscyamine hydrobromide, 500 μl catalase [13,18]. The reaction was started by the addition of the crude protein extract and was incubated at 30°C for 15 h. All the glass materials used were treated with HNO_3 _to eliminate traces residual metals and the experiments were carried out by triplicate.

### Alkaloid extraction

The alkaloid extraction of the reaction mixture mentioned above was performed at pH 9. Samples were extracted three times with 5 ml chloroform by vortexing and the organic phase was evaporated under gaseous N_2_. The residue was dissolved in methanol-water (50:50 v v^-1^) and filtered trough a 0.45 μm pore nylon membrane.

### Analytical Methods

HPLC analysis of the alkaloids was performed on a Shimadzu LC-20AT system with a LiChro CART column 125–4 Lichrospher 60 RP-select B (5 μm), Merck (Darmstadt, Germany). The elution was performed at 40°C, isocratically with Octanesulfonic acid 0.01 M pH3/Metanol (65:35 v v^-1^) at a 1 ml min^-1 ^flow rate. The detection was performed at 220 nm.

## Competing interests

The authors declare that they have no competing interests.

## Authors' contributions

ABC, JRT and AMG conceived, designed the experiments and analyzed the data, ABC performed all the experiments. All authors wrote the paper and approved the final version of the manuscript.
